# Case Report: Identification of a *HNF1A* exons 1–10 heterozygous deletion in a Chinese MODY family

**DOI:** 10.3389/fendo.2026.1743021

**Published:** 2026-02-16

**Authors:** Mengyun Lei, Mei Xue, Huawei Wang, Zhe Dai, Jun Tang

**Affiliations:** Department of Endocrinology, Zhongnan Hospital of Wuhan University, Wuhan, China

**Keywords:** HNF1A, HNF1A-MODY, MLPA, MODY, MODY3

## Abstract

**Background:**

Maturity-onset diabetes of the young (MODY) is an autosomal dominant monogenic diabetes, with HNF1A-MODY (MODY3) being a common subtype. Standard genetic testing for MODY often focuses on sequencing, which can lead to the misdiagnosis of cases caused by *HNF1A* copy number variants (CNVs). This study investigates the diagnosis of a Chinese family with a *HNF1A*(NM_000545.8):ex1_10del.

**Methods:**

We evaluated a Chinese family with a clinical diagnosis of maturity-onset diabetes of the young (MODY). Clinical data and peripheral blood samples were collected from family members. A heterozygous *HNF1A*(NM_000545.8):ex1_10del was suspected by next-generation sequencing (NGS) using a hereditary diabetes gene panel.This finding was validated using multiplex ligation-dependent probe amplification (MLPA). We also conducted a literature review of previously reported HNF1A-MODY cases associated with heterozygous exon deletions.

**Results:**

A heterozygous *HNF1A*(NM_000545.8):ex1_10del was identified by MLPA in the pedigree after next-generation sequencing (NGS) detected no pathogenic single-nucleotide variants (SNVs) or small insertions/deletions (indels). The deletion was classified as pathogenic according to ACMG/AMP and ClinGen guidelines. The family’s clinical phenotype aligned with previously reported HNF1A-MODY cases caused by whole-gene or exon deletions, showing similarities to phenotypes associated with SNVs and small indels. Following genetic diagnosis, the proband was transitioned from insulin to glimepiride, achieving optimal glycemic control.

**Conclusions:**

This study identifies a *HNF1A* whole-gene deletion in a Chinese family with MODY, confirming the effectiveness of sulfonylureas for HNF1A-MODY management. Large *HNF1A* deletions, undetectable by standard sequencing, can cause MODY and necessitate copy number variant (CNV) analysis. MLPA is essential for definitive MODY diagnosis, particularly in cases with strong clinical suspicion but negative sequencing results. These findings broaden the known spectrum of *HNF1A* mutations and highlight the critical role of CNV detection in MODY genetic testing.

## 1 Introduction

Maturity-onset diabetes of the young (MODY) comprises a heterogeneous group of monogenic diabetes syndromes characterized by early-onset hyperglycemia, typically diagnosed before 25 years of age, and most inherited in an autosomal dominant manner (1). Although MODY accounts for only approximately 1-2% of all diabetes cases in population-based estimates (2), its true prevalence is likely underestimated due to frequent misclassification as type 1 or type 2 diabetes (3).This under-recognition is clinically important because an accurate molecular diagnosis of MODY directly determines optimal treatment, prognosis, and the need for predictive genetic testing in relatives.

MODY is caused by pathogenic variants in genes involved in pancreatic β-cell development, glucose sensing, and insulin secretion. To date, pathogenic variants in more than 11 genes have been definitively linked to MODY (4). The most common subtypes, accounting for over 90% of genetically confirmed cases, are caused by mutations in *GCK* (MODY2), *HNF1A* (MODY3), and *HNF4A* (MODY1) (4). *GCK*, located on chromosome 7p15.3, encodes glucokinase, a hexokinase that catalyzes the phosphorylation of glucose in pancreatic β-cells and hepatocytes and serves as a critical glucose sensor (5). *GCK* contains multiple promoters and alternative transcript isoforms, with dominant expression in liver and pancreatic islets. Pathogenic GCK variants reduce glucose-stimulated insulin secretion, resulting in lifelong, mild, stable fasting hyperglycemia (5). *HNF1A*, located on chromosome 12q24.31 and composed of 10 exons, encodes a homeodomain-containing transcription factor that binds DNA as a homodimer or heterodimer to regulate the expression of liver- and β-cell–specific genes, including those involved in glucose transport and insulin secretion (6). *HNF4A*, located on chromosome 20q13.12 and consisting of 13 exons, encodes a nuclear receptor transcription factor that directly regulates *HNF1A* expression and controls a broad hepatic and pancreatic transcriptional program (7). *HNF1B*, located on 17q12, encodes a related transcription factor that forms homo- and heterodimers with *HNF1A* and plays a crucial role in renal and pancreatic development (8). Other MODY-associated genes include *INS* (MODY10) on 11p15.5, which encodes the insulin prohormone; *PDX1* (MODY4) on 13q12.2, and *NEUROD1* (MODY6) on 2q31, both of which encode essential β-cell transcription factors regulating insulin gene transcription and islet differentiation (4).

Clinically, MODY exhibits a broad phenotypic spectrum. GCK-MODY typically presents as lifelong, mild, non-progressive fasting hyperglycemia that is often incidentally discovered, including during pregnancy screening. These patients rarely develop microvascular complications and usually do not require pharmacologic glucose-lowering therapy outside of pregnancy (4, 9). In contrast, HNF1A-MODY and HNF4A-MODY are characterized by progressive β-cell dysfunction. Patients typically present in adolescence or early adulthood with increasing hyperglycemia, marked postprandial glucose excursions, and a low renal threshold for glucose, resulting in prominent glycosuria (4, 9). Endogenous insulin secretion is usually preserved early in the disease course but declines progressively over time. HNF4A-MODY additionally shows distinctive neonatal features, including transient hyperinsulinemic hypoglycemia and large-for-gestational-age birth weight (4, 9).

The diagnosis of MODY requires a high index of clinical suspicion and integration of phenotypic, biochemical, and family history data. Typical features include early-onset diabetes, autosomal dominant inheritance across successive generations, absence of pancreatic autoantibodies, preserved C-peptide levels indicating endogenous insulin production, lack of obesity or severe insulin resistance (4, 9). Clinical tools such as MODY probability calculators may aid in selecting candidates for genetic testing, but definitive diagnosis relies on molecular genetic testing, preferably with NGS-based monogenic diabetes panels followed by segregation analysis (4). MODY represents a classical model of precision medicine because treatment can be directly guided by genotype. GCK-MODY generally requires no glucose-lowering therapy outside pregnancy. In contrast, patients with HNF1A-MODY and HNF4A-MODY are characteristically highly sensitive to low-dose sulfonylureas, which are effective as first-line therapy in most cases, particularly early in the disease course. Many patients can successfully transition from insulin to sulfonylureas without increased risk of ketoacidosis. With progressive β-cell failure, some individuals eventually require insulin therapy (4, 9).

HNF1A-MODY is caused by pathogenic variants in the HNF1A gene encoding a key transcription factor involved in pancreatic β-cell function and glucose metabolism. These variants impair downstream transcriptional programs regulating insulin secretion, leading to progressive β-cell failure (6, 10). HNF1A-MODY demonstrates high but age-dependent penetrance, with approximately 60–65% of carriers developing diabetes by 25 years of age and over 90% by mid-adulthood (11). Previous studies indicate that HNF1A-MODY is present but relatively uncommon in Chinese populations. Early family-based investigations identified HNF1A mutations in approximately 9% of MODY-like pedigrees, including several novel variants. More recent cohorts report a frequency of about 15–16% among genetically confirmed MODY cases. However, in broader young-onset diabetes populations, the prevalence is substantially lower (~0.2–0.3%). Overall, the mutation spectrum appears heterogeneous—with both inherited and *de novo* variants—but the overall frequency remains lower than that reported in European populations (12–14).

Molecular testing for MODY typically involves sequencing of known MODY genes. However, copy‐number variants (CNVs), such as large multi−exon deletions, can pose a diagnostic challenge. Large deletions in *HNF1A* or *GCK* can cause MODY but are not detected by standard PCR−based sequencing methods; they require gene−dosage assays such as multiplex ligation−dependent probe amplification (MLPA) (15). Here we report a 32−year−old Chinese male with early−onset, antibody−negative diabetes who was found to have a heterozygous HNF1A(NM_000545.8): ex1_10del. We detail his clinical presentation, laboratory and biochemical findings, and genetic results, and compare this case with previously reported *HNF1A* deletion cases. We discuss the diagnostic and therapeutic implications, highlighting lessons on precision medicine for monogenic diabetes.

## 2 Case presentation

**Figure 1:**
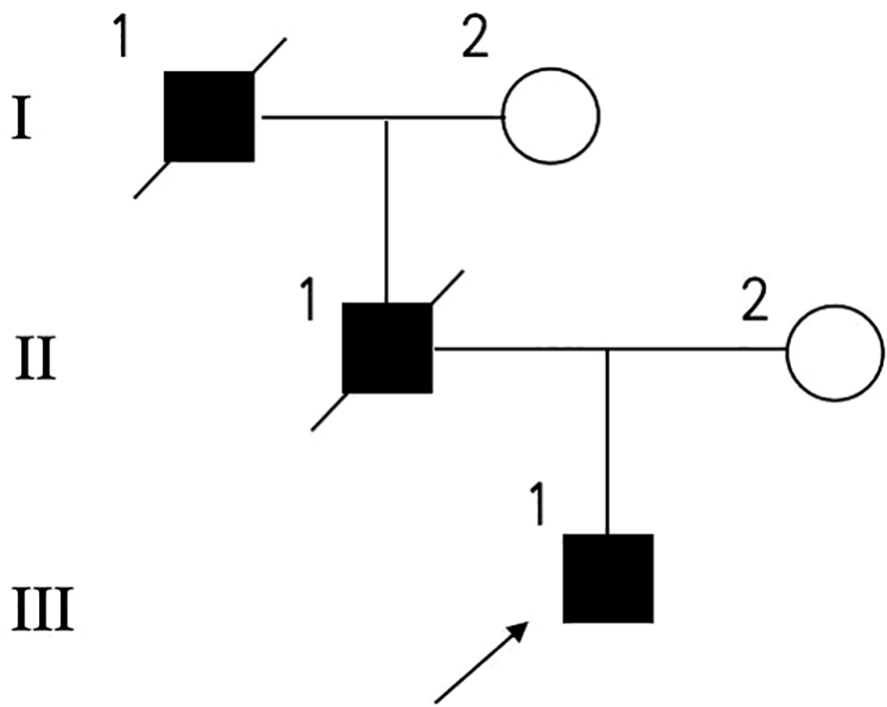
Pedigree of the family. Circles represent females, and squares represent males, and a diagonal line through a symbol indicates that the individual is deceased. Filled symbols indicate diabetic individuals and proband is shown by an arrow.

The proband (III-1, **Figure 1**), a 32-year-old man, was diagnosed with diabetes at the age of 24 due to a two-year history of polydipsia and polyuria. At the time of evaluation, the body mass index (BMI) was 18.8 kg/m², indicating a lean body type. The HbA1c level was 8.7%, suggesting poor long-term glycemic control. Pancreatic autoantibodies were negative, indicating the absence of autoimmune diabetes. Renal function showed normal blood urea but slightly decreased creatinine and uric acid; electrolytes, β-hydroxybutyric acid, inflammatory markers, and hematological parameters were within reference ranges. Urine glucose was strongly positive (++++), while urine ketones were negative (**Table 1**). The results of the oral glucose tolerance test (OGTT) are summarized in **Table 2**. The fasting blood glucose was 6.27 mmol/L, which increased sharply to 21.94 mmol/L at 2 hours and remained elevated (17.38 mmol/L) at 3 hours, indicating impaired glucose tolerance and poor glycemic regulation. C-peptide and insulin levels both showed an initial increase following glucose load, peaking at 1 hour (C-peptide: 4.01 ng/mL; insulin: 15.3 μIU/mL) and gradually declining thereafter (C-peptide: 2.96 ng/mL; insulin: 12.5 μIU/mL at 3 hours). Although insulin and C-peptide secretion increased in response to glucose stimulation, the levels were relatively low in comparison to the marked hyperglycemia, suggesting an inadequate β-cell secretory response.

**Table 1:** Clinical features and laboratory test results of the proband.

Parameter	Result	Reference value
Age (years)	32	
Age at diagnosis (years)	24	
BMI (kg/m2)	18.8	
HbA1C mmol/mol (%)	72 (8.7%)	
GAD-Ab	Negative	Negative
IA-Ab	Negative	Negative
ICA-Ab	Negative	Negative
IA-2-Ab	Negative	Negative
Blood Urea (mmol/L)	5.23	2.8-7.6
Creatinine (umol/L)	59.5	64-104
Uric Acid (umol/L)	205.4	208-428
Potassium (mmol/L)	3.81	3.5-5.3
Sodium (mmol/L)	140.2	137-147
β-Hydroxybutyric Acid (mmol/L)	0.08	0-0.28
C-Reactive Protein (mg/L)	1.0	0-10
White Blood Cell Count (10^9/L)	7.91	3.5-9.5
Hemoglobin (g/L)	167.3	130-175
Platelet Count (10^9/L)	234	125-350
Prothrombin Time (s)	11.6	9.4-12.5
D-Dimer (ng/ml)	18	0-500
Urine glucose	++++	Negative
Urine ketone	Negative	Negative

**Table 2:** Results of blood glucose, C-peptide and insulin in oral glucose tolerance test (OGTT).

Time	Blood Glucose (mmol/L)	C peptide (ng/ml)	Insulin (uIU/ml)
0h	6.27	1.18	2.57
0.5h	13.67	2.82	9.09
1h	17.97	4.01	15.3
2h	21.94	3.82	16.1
3h	17.38	2.96	12.5

The patient’s family history was significant for diabetes on the paternal side (**Figure 1**). His father (II-1) was diagnosed with diabetes at age 42 and was reportedly managed as type 2 diabetes; he later developed diabetic nephropathy and ultimately died from end-stage renal disease. The patient’s paternal grandfather also had diabetes and died from related complications. The patient’s mother (II-2) has no history of diabetes, and no other relatives were available for genetic testing. Given the young age at onset, negative autoantibodies, preserved BMI, low C-peptide, and a suggestive family history (father and grandfather with diabetes), monogenic diabetes was suspected (1).

**Figure 2:**
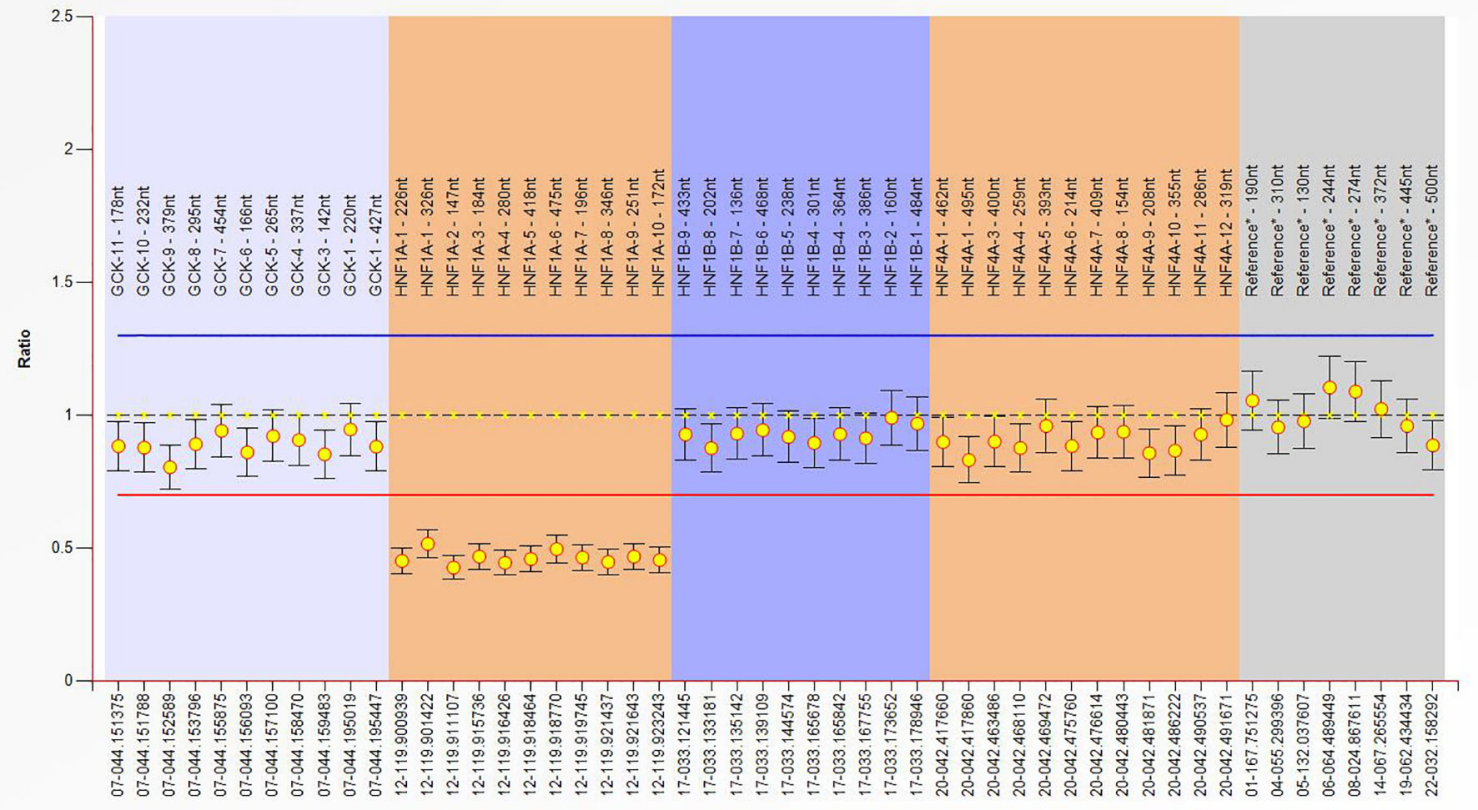
Detection of a heterozygous *HNF1A*(NM_000545.8):ex1_10del by MLPA.

Genetic testing by NGS identified no pathogenic single nucleotide variants (SNVs) or insertions/deletions (indels) in any known MODY genes, suggesting the possible presence of a large deletion in the *HNF1A* gene. We then conducted MLPA analysis targeting the *HNF1A* gene, which identified a heterozygous *HNF1A*(NM_000545.8): ex1_10del. (**Figure 2**). According to ACMG criteria, this deletion is considered pathogenic, confirming the diagnosis of HNF1A-MODY.

Based on this molecular diagnosis, the patient’s treatment was changed from insulin to an oral sulfonylurea. Insulin (mealtime aspart 8 units) was gradually withdrawn, and glimepiride was initiated at 1 mg daily. Within three months of switching to glimepiride alone, the patient’s blood glucose levels normalized. At follow-up one year later, his HbA1c had improved to 6.7% on glimepiride monotherapy, with no hypoglycemic episodes reported. This excellent response to low-dose sulfonylurea is consistent with the known pharmacogenetic signature of HNF1A-MODY (6, 10).

We summarized two representative reports of *HNF1A* exon or whole-gene deletions associated with HNF1A-MODY (**Table 3**). The first report by Ellard et al (15). described a cohort of probands screened using a custom MLPA assay covering GCK, HNF1A, and HNF4A (30 exons total). Multiple deletion patterns were identified in different probands, including HNF1A(NM_000545.8):ex1del, ex2_10del, and ex1_10del. Familial segregation analysis confirmed co-segregation of the deletions with early-onset diabetes. In this cohort, 4 out of 60 (6.7%) HNF1A-negative probands carried large deletions, highlighting the importance of gene dosage analysis in MODY diagnosis.Willson et al. (16) reported an in-frame *HNF1A*(NM_000545.8):ex2_3del in a HNF1A-MODY family, initially detected by array-CGH and confirmed by direct sequencing. The deletion co-segregated in three affected family members, all of whom developed both HNF1A-MODY and hepatocellular adenomas, indicating an extended phenotype beyond diabetes. These studies collectively demonstrate that *HNF1A* deletions may present with HNF1A-MODY and, in some cases, hepatic adenomas, underscoring the clinical relevance of CNV testing when sequencing alone is negative.

**Table 3:** Summary of representative reports describing *HNF1A* exon or whole-gene deletions.

No.	Reference	Exon(s) deleted	Detection and validation method	Family segregation	Clinical and phenotypic correlation
1	Ellard S et al. (15)	ex1del, ex2_10del, ex1_10del. (different probands)	Custom MLPA assay using synthetic oligonucleotide probes covering GCK, HNF1A, and HNF4A (30 exons total)	Co-segregation with early-onset diabetes confirmed within families	4/60 (6.7%) HNF1A-negative probands had large deletions; emphasizes need for dosage analysis in MODY diagnosis
2	Willson JSB et al. (16)	ex2_3del	Detected by array-CGH and confirmed by direct sequencing	Three affected family members carried same deletion (HNF1A-MODY + hepatocellular adenoma)	Coexistence of HNF1A-MODY and hepatocellular adenoma/HCC; suggests extended phenotype

## 3 Materials and methods

### 3.1 Clinical evaluation

We obtained a detailed history and conducted a physical examination. Height, weight, and body mass index (BMI) were recorded. The family history of diabetes was documented through interviews with the patient and relatives.

### 3.2 Laboratory testing

OGTT and glycated hemoglobin (HbA_1_c) were measured. Pancreatic autoantibodies – including anti-human Insulin Antibody, anti-Glutamic Acid Decarboxylase Antibody, anti-Pancreatic Islet Cell Antibody, anti-Tyrosine Phosphatase Antibody. Additional workup included renal function tests, liver enzymes, lipid profile, and screening for microvascular complications (fundoscopic exam, neuropathy, and albuminuria).

### 3.3 Genetic analysis

Genomic DNA was extracted from peripheral blood leukocytes using standard protocols. For the identification of candidate variants, we first performed next-generation sequencing (NGS) using a targeted monogenic diabetes gene panel, which included *HNF1A*, *GCK*, *HNF4A*, *HNF1B*, *PDX1*, and other known MODY-associated genes. Sequencing libraries were prepared following the manufacturer’s instructions, and the NGS workflow included rigorous quality control metrics, with rigorous evaluation of sequencing quality metrics, including minimum read depth per targeted region, base-calling accuracy scores, and evenness of coverage across all exons, to guarantee robust and reproducible variant identification. Variants were called using validated bioinformatics pipelines and interpreted according to the American College of Medical Genetics and Genomics (ACMG)/Association for Molecular Pathology (AMP) guidelines for pathogenicity.

We performed multiplex ligation-dependent probe amplification (MLPA) to detect potential large deletions or duplications. Custom MLPA probes were designed to cover all 10 coding exons of *HNF1A*. MLPA assays were performed following standard protocols, and data quality was assessed based on probe signal intensity, inter-probe consistency, and reference sample comparison to ensure accurate detection of copy number variations.

All participants provided written informed consent, and the study was approved by the Medical Ethics Committee of Zhongnan Hospital of Wuhan University (No: 2023029).

## 4 Discussion

This case demonstrates that a heterozygous *HNF1A*(NM_000545.8):ex1_10del can cause a classic HNF1A-MODY phenotype. The clinical features – early-onset diabetes, low BMI, negative autoantibodies, low C-peptide indicating β-cell failure, and a multigenerational family history – strongly resembled those of MODY cases (1). Importantly, despite the unusual mutation type (a whole-gene deletion), the clinical phenotype did not differ qualitatively from that seen with point mutations or small indels in *HNF1A*. Like many HNF1A-MODY patients, our proband had progressive β-cell failure yet achieved excellent glucose control on an oral sulfonylurea. Our patient’s response to low-dose glimepiride – achieving HbA1c of 6.7% is consistent with the known sulfonylurea sensitivity of HNF1A-MODY (6). Since the genetic defect impairs glucose-stimulated insulin secretion upstream of sulfonylurea action (6). Over time, patients with HNF1A-MODY typically maintain good control on sulfonylureas, although progressive β-cell decline may eventually necessitate insulin.

*HNF1A* encodes a homeodomain-containing transcription factor crucial for regulating genes in pancreatic β cells (as well as in the liver and kidney) (6, 10). Haploinsufficiency of *HNF1A* leads to reduced transcription of insulin and other β-cell genes, resulting in impaired glucose-stimulated insulin secretion (6, 10). Furthermore, HNF1A regulates renal glucose handling; loss of one allele lowers the renal threshold for glucose reabsorption, causing glycosuria at lower blood glucose levels (17). Although we did not formally measure the renal threshold, the presence of polyuria despite only modest hyperglycemia in our patient suggests the renal glycosuria typical of HNF1A-MODY. The *HNF1A*(NM_000545.8):ex1_10del eliminates the entire coding sequence, effectively creating a null allele. Yet the resulting phenotype in our patient (and others with this deletion) is strikingly like that of patients with missense or smaller truncating mutations in HNF1A. This underscores that the phenotype of HNF1A-MODY is driven by the loss of HNF1A function, regardless of mutation type.

Multi-exon *HNF1A* deletions can underlie HNF1A-MODY and are often missed by standard sequencing, highlighting the critical role of CNV analysis (15). Although whole-gene or multi-exon deletions are most commonly associated with HNF1B-MODY (18), they also occur in *HNF1A* and *GCK (*15). In our patient, MLPA identified a *HNF1A* exons 1–10 deletion after panel sequencing was uninformative; without gene dosage analysis, the molecular diagnosis would have been missed, potentially leading to unnecessary insulin therapy. Prior studies support this approach: Ellard et al. detected *HNF1A* deletions in 6.7% of sequencing-negative HNF1A -MODY cases and recommended routine MLPA (15), and current guidelines likewise advocate including dosage analysis in MODY gene testing (19).

Beyond glycemic management, *HNF1A* mutations can have extrapancreatic manifestations. In particular, hepatic adenomas are reported in some patients with HNF1A-MODY (16, 20, 21). Although our patient currently has normal liver imaging and function tests, periodic abdominal surveillance has been arranged given this risk. Hepatic adenomas should be monitored due to potential hemorrhage or rare malignant transformation. These findings reinforce that comprehensive genetic testing, including CNV assessment, is essential not only for accurate HNF1A-MODY diagnosis and tailored diabetes management but also for anticipating extrapancreatic complications such as hepatic adenomas.

In summary, we identified a heterozygous *HNF1A*(NM_000545.8):ex1_10del in a Chinese patient with early-onset, antibody-negative diabetes, confirming HNF1A-MODY. The clinical features—including strong family history, sulfonylurea responsiveness, and absence of obesity or autoimmunity—were typical of HNF1A-MODY despite the atypical genetic lesion. This case highlights the importance of comprehensive genetic testing for MODY, which should include CNV analysis (e.g., MLPA) in patients with negative sequencing results. Early molecular diagnosis allowed tailored therapy, switching from insulin to low-dose sulfonylurea, and informed family screening and periodic surveillance for hepatic adenomas, exemplifying precision medicine in diabetes care.

## Data Availability

The original contributions presented in the study are included in the article. The raw sequence data reported in this paper have been deposited in the Genome Sequence Archive in National Genomics Data Center, China National Center for Bioinformation / Beijing Institute of Genomics, Chinese Academy of Sciences (GSA-Human: HRA016657) that are publicly accessible at https://ngdc.cncb.ac.cn/gsa-human.
